# Selection on the joint actions of pairs leads to divergent adaptation and coadaptation of care-giving parents during pre-hatching care

**DOI:** 10.1098/rspb.2024.0876

**Published:** 2024-06-12

**Authors:** Benjamin J. M. Jarrett, Rahia Mashoodh, Swastika Issar, Sonia Pascoal, Darren Rebar, Syuan-Jyun Sun, Matthew Schrader, Rebecca M. Kilner

**Affiliations:** ^1^ School of Environmental and Natural Sciences, Bangor University, Bangor, UK; ^2^ Department of Zoology, University of Cambridge, Cambridge, UK; ^3^ Department of Biology, Lund University, Lund, Sweden; ^4^ Department of Biological Sciences, Emporia State University, Emporia, KS, USA; ^5^ International Degree Program in Climate Change and Sustainable Development, National Taiwan University, Taipei, Taiwan; ^6^ Department of Biology, University of the South, Sewanee, TN, USA

**Keywords:** nest-building, reproductive isolation, division of labour

## Abstract

The joint actions of animals in partnerships or social groups evolve under both natural selection from the wider environment and social selection imposed by other members of the pair or group. We used experimental evolution to investigate how jointly expressed actions evolve upon exposure to a new environmental challenge. Our work focused on the evolution of carrion nest preparation by pairs of burying beetles *Nicrophorus vespilloides*, a joint activity undertaken by the pair but typically led by the male. In previous work, we found that carrion nest preparation evolved to be faster in experimental populations without post-hatching care (No Care: NC lines) than with post-hatching care (Full Care: FC lines). Here, we investigate how this joint activity evolved. After 15 generations of experimental evolution, we created heterotypic pairs (NC females with FC males and NC males with FC females) and compared their carrion nest making with homotypic NC and FC pairs. We found that pairs with NC males prepared the nest more rapidly than pairs with FC males, regardless of the female’s line of origin. We discuss how social coadaptations within pairs or groups could act as a post-mating barrier to gene flow.

## 1. Introduction

When animals interact in partnerships, families or social groups, they are often in conflict, for example, over mating [1], parental care [2] or who gets to reproduce [3]. But, when the conflict is at least partially resolved or suppressed, their joint activities can enhance the fitness of the whole collective [4]. Joint actions commonly function to overcome ecological adversity in the wider environment (e.g. [5]), such as the threat of attack by predators (e.g. [6]), the potential theft of a key resource by rivals (e.g. [7]) or the patchy availability of nutritional resources (e.g. [8]). It might involve the joint construction of a nest or communal burrow (e.g. [9]), for example, or collective defence against pathogens (e.g. [10]) or group care of dependent offspring (e.g. [7]). Working together to achieve a common goal favours individuals whose activities are well-coordinated with the actions of their partner, or other members of the family or social collective (e.g. [11]), perhaps through division of labour (e.g. [12]). It means that for each individual in the collective, there are two inter-related sources of selection on their behaviour: (i) natural selection from ecological challenges in the wider environment that the team is working collectively to overcome and (ii) social selection from the other members of the pair or group to fine-tune individual contributions to the pair or group’s collective activities [11–14].

Theory predicts that adapting to the heritable environments created by social partners can be more rapid than adaptation to other environments. Adaptation to the social environment, which has a genetic and heritable component, changes the frequency of the same social environment and thus has the potential to reinforce selection in the subsequent generations, in a positive feedback loop [15–17]. Furthermore, the social coadaptation of the different group members establishes favourable combinations of phenotypes within the family. These are known to exist within animal families (e.g. [18–20]), particularly among provisioning adults who coordinate their activities very precisely (e.g. [21–23]). Favourable phenotypic combinations potentially result from intergenomic epistasis in which the fitness of a social trait depends on the genotype of the social partner [24,25].

The twin sources of selection on social traits from the environment and social partners make it difficult to predict how social traits might evolve upon exposure to a new ecological challenge in the wider environment. Would all individuals in the pair or group experience the same selection pressures in response to a new ecological challenge in the first instance, and only secondarily respond to selection induced by their behaviour to each other? Or would selection act on a subset of the population who first adapt to ecological change, and their new actions then provoke new social coadaptations in other members of the pair or group? The latter scenario seems more likely for social activities in which there are pre-existing leaders and followers (e.g. [26]), or where labour is already unevenly divided (e.g. [11,20,27,28]), though whether this is indeed what happens has seldom been tested experimentally.

We investigated this problem by establishing lines of burying beetles *Nicrophorus vespilloides* and allowing them to evolve in replicate experimental environments in which we either left parents to care for their offspring after hatching or removed post-hatching parental care. Removing care created an ecological challenge for the orphaned offspring who were suddenly exposed to a wider environment from which parents would otherwise have shielded them, and presented a social challenge by placing parents under selection to adapt their pre-hatching behaviour to each other [29]. Burying beetles breed on the body of a small dead vertebrate [30], commonly in pairs but sometimes as lone adults or multi-adult groups [31]. The parents jointly convert the dead mouse or bird into a carrion nest by removing the fur or feathers, rolling it into a ball, covering it in anti-microbial anal exudates and burying it. Eggs are laid in the soil surrounding the sunken carrion nest, and newly hatched larvae crawl to the nest where they aggregate. To help their larvae take up residence on the carrion nest, and feed upon it, parents may make an incision in the flesh prior to their arrival. The joint nest preparation activities of the parents are the focus of interest here. Parents also guard and feed larvae after they hatch, though larvae can survive in the laboratory with no post-hatching care at all [32].

In wild populations, pairs are under selection to prepare and bury the carrion nest as effectively and efficiently as possible. Carrion nest preparation is key for concealing this vital resource from rivals who might wish to use it for their own reproduction, including blowflies [33] and other carrion-breeding insects, congenerics and conspecifics and microbes [30]. We imposed selection on the pair’s joint carrion preparation activities by removing parents 53 h after pairing, just before their offspring started to hatch (the two replicate No Care lines; hereafter 'NC'). Parents in the NC lines were consequently unable to provide any post-hatching care, though they were able to perform pre-hatching care behaviours. In two other experimentally evolving lines, parents were allowed to stay with their larvae throughout their development and so were able to provide both pre-hatching and post-hatching parental care (the two replicate Full Care lines; hereafter 'FC').

During the first 20 generations of experimental evolution, the NC lines adapted rapidly to our experimental intervention [34,35] and exhibited divergent phenotypes in both larval [36–38] and parental traits [29,36,39]. Crucially, all the adaptations to a life without parental care that occurred within 20 generations of experimental evolution were consistent between replicate populations, including the timing of making the feeding incision [29,36]. Of particular relevance here, NC parents evolved to frontload their parental care. They became more effective at converting carrion into a nest for their larvae, accelerating the pace of carrion nest preparation, and in this way increased their offspring’s chance of surviving without post-hatching care [29]. In particular, NC parents were more likely to make an incision into the carcass prior to larval hatching. This incision is noticeable upon inspecting the carcass, its creation is one of the final acts of carrion nest preparation, and its presence prior to larval hatching strongly predicts brood success when larvae are deprived of any post-hatching care [29,32,36].

In common with many other species [40], burying beetles divide the duties of parental care between the sexes. Males carry out more of the pre-hatching duties of care and take the lead in preparing the carrion nest [41,42], while females are more involved with direct post-hatching care [41]. Previous work on burying beetles suggests that task specialization causes the sexes to be dependent upon each other, in the sense that they each perform more effectively when paired [43]. After 15 generations of experimental evolution, we conducted crosses between the experimental lines to identify the agents of selection acting on parents. We considered two possibilities: (i) that a parent had independently adapted its nest preparation behaviour to the NC condition; and/or (ii) that a parent had adapted to the behaviour of its partner from the same lineage. To distinguish between these two alternatives, we generated homotypic pairings (NC adults paired with each other and FC adults paired with each other) and heterotypic pairings (NC adults paired with FC adults). We also used these data to investigate whether the presence of a feeding incision predicted the survival of the brood when parents were present or absent after hatching and whether broods produced by heterotypic pairs were less likely to survive when parents were absent.

We predicted that if males had independently adapted to an NC environment (because they take the lead in carrion preparation), then nests prepared by NC males should consistently bear a feeding incision with a higher probability than carrion prepared by FC males—regardless of the female’s line of origin. If the NC females had coadapted to the NC males, then carrion prepared by NC females paired with NC males should be more likely to carry an incision 53 h after pairing than carrion prepared by NC females paired with FC males. By contrast, if the sexes had each independently evolved changes in their nest preparation behaviour in the NC lines, then the FC homotypic pairs should be least likely to bear a feeding incision 53 h after pairing, while the NC homotypic pairs should be most likely to have an incision, and both heterotypic pairs should lie somewhere between the two.

## 2. Methods

### (a) Burying beetle husbandry

Adult *N. vespilloides* beetles were kept individually in boxes measuring 12 × 8 × 2 cm filled with compost for two weeks after eclosion until they were sexually mature. Individuals were fed ∼0.3 g of minced beef twice a week. Once individuals were sexually mature, they were paired for breeding. The pair of beetles was added to a larger box measuring 17 × 12 × 6 cm half filled with fresh compost. A mouse carcass, sourced from Live Foods Direct, was weighed and recorded (mean ± s.d. = 11.60 ± 0.87 g; range = 10.00–13.18 g) and placed into the box, after which the pair of beetles was added. The pair of beetles prepared the mouse carcass for the first two days, while the female laid eggs in the soil surrounding the carcass. At ∼53 h, when carcass preparation and egg laying had been completed, we removed the parents in the NC treatment. For the FC treatment, the parents were left in the box until the larvae dispersed, typically eight days after pairing.

Eight days after pairing, the larvae had completely eaten the carcass and were ready to complete development. We removed the larvae, counted them and weighed the whole brood to the nearest 0.0001 g. After they had been weighed, the larvae were placed into an eclosion box, measuring 10 × 10 × 2 cm, with 25 individual cells, each 2 × 2 × 2 cm. An individual larva was placed in each cell and covered with peat that was sifted to remove large chunks of soil. Each box held one brood. Water was sprayed over the top to prevent desiccation during subsequent development, which typically lasted 18–21 days.

### (b) Experimental evolution

The experimentally evolving lines used in this work have been described in detail elsewhere [35]. In brief, we established a large founding population of 671 *N*. *vespilloides* individuals by interbreeding wild-caught individuals from four different woodlands in 32 pairs. Only one pair of the 32 comprised individuals from the same woodland population. Offspring from each of the 32 pairs were represented in each of the four replicate experimental lines. In two lines, larvae experienced FC in each generation, with both parents allowed to stay in the breeding box to feed and interact with their offspring. In the remaining NC lines, both parents were removed from the breeding box at each generation, 53 h after they were paired, once carcass preparation was complete but before any larvae had hatched (see [35] for more details on the experimental set-up). Pairs only bred once and only had one partner. FC lines had 30 pairs of breeding per generation, and NC lines had 45 pairs to account for failures. Siblings and cousins were prevented from breeding. The lines were organized into two blocks, separated by a week, to ease the workload (hence NC1, FC1 in Block 1 and NC2, FC2 in Block 2). The experimental work reported here used lines that had been exposed to 15 generations of experimental evolution.

### (c) Experimental design

**Figure 1.**
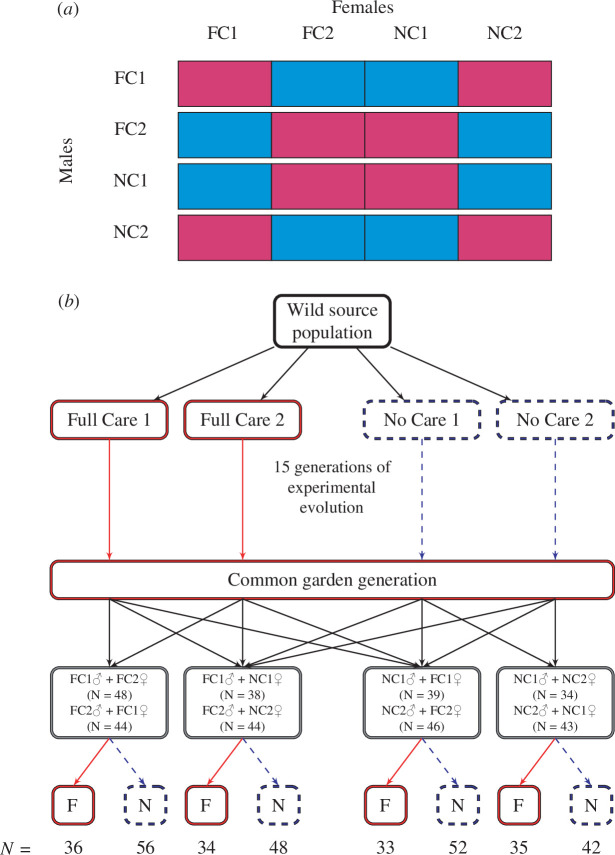
(*a*) Experimental design, showing all possible crosses between lines, including all replicate lines within parental care treatments. Blue boxes indicate the crosses that were made, and red boxes indicate the crosses that were not made. FC = Full Care, NC = No Care; numbers refer to replicate lines. (*b*) The experimental design is in detail. Four genetically similar lines were generated from a single source population that was split into two replicate FC (in red) and two replicate NC (in blue) lines [34]. The FC lines evolved in an FC environment (red solid lines) and the NC lines evolved in an NC environment (blue dashed lines) for 15 generations (refer [34] for more information about the experimental set-up). After 15 generations of breeding in these contrasting environments, individuals from each population were passed through a common garden regime, in which all broods received FC to minimize variation between lines owing to parental effects. The populations were then crossed. The two control groups involved crossing the populations that had evolved under the same social environment during development: FC pairs were formed by males and females from FC1 and FC2; and NC pairs were formed by males and females from NC1 and NC2. The two experimental groups involved crossing populations that have evolved under different social environments during development. We only crossed one FC line with its NC replicate (FC1 with NC1, and FC2 with NC2) for both sets of crosses. The offspring from each pair were then randomly allocated one of the social environments (FC or NC) as a treatment during larval development.

The design of the experiment is shown in figure 1. Prior to testing, the four lines went through a common garden environment of FC (where all parents were allowed to interact with their larvae throughout development). The aim was to standardize parental effects across all four treatments so that any residual variation could be attributed to evolutionary change. To set-up the common garden generation, the FC1 and NC1 parents were bred three weeks after eclosing as adults, and the FC2 and NC2 parents were bred at two weeks after eclosing as adults. This synchronized breeding of individuals from the two blocks ensured that the adults generated for the focal experiment were matched in age at first breeding across treatments. The lines were then crossed: homotypic pairs involved crossing the two replicate lines that had experienced the same parental manipulation (i.e. FC1 × FC2 and NC1 × NC2, figure 1*a*,*b*), while heterotypic pairs involved crossing populations that had experienced different parental manipulations (i.e. FC1 × NC1 and FC2 × NC2, figure 1*a*,*b*). It was not feasible logistically to perform every possible cross between every replicate line (figure 1*a*). With the experimental design we used, we can isolate the evolutionary effects of parental care treatment (NC versus FC) on males and females (figure 1*a*), but we cannot partition effects of replicate line (1 versus 2) from parental care treatment (NC versus FC). Note that when we carried out this experiment, we detected no evidence of divergence between replicate lines within parental care treatments.

### (d) The adaptive value of the feeding incision

We also investigated the fitness consequences of the feeding incision by asking how its presence or absence influenced the likelihood that a brood would succeed (i.e. that the brood would have at least one surviving larva at dispersal) and, if the brood was successful, how many larvae survived to disperse from the carcass (‘brood size’). We anticipated that the relationship between the feeding incision and brood success (or brood size) would be dependent on the care environment, and so split the NC and FC environment data and analysed each separately. For this analysis, we analysed brood success (success versus fail) assuming a Bernoulli distribution and brood size using a negative binomial distribution. The model included male line of origin (i.e. descended from an NC or FC line) and female line of origin (i.e. descended from an NC or FC line) and their interaction, along with the presence or absence of a feeding incision as a two-level factor.

### (e) Adaptation and coadaptation

We tested whether the lines of origin of both males and females each independently predicted the presence of a feeding incision on a carcass. We coded males and females from the FC lines as 1 and individuals from the NC lines as 0. The model included both the female and male lines of origin and their interaction. Simpler models were then considered by first removing the interaction between female and male lines of origin, then second removing each line of origin in turn and finally considering a model without either line of origin. For all analyses, the standardized (mean = 0, s.d. = 1) mass of the carcass, and the standardized size of the male and the female were included as covariates. Other covariates included in specific models are outlined below.

### (f) Statistical analysis

All analyses were performed in R (version 3.5.1) [44] using Bayesian models in Stan [45] implemented in *brms* [46,47]. Additional packages used for the analysis and plotting of data in this paper include: *tidyverse* [48], *ggplot2* [49], *tidybayes* [50] and *modelr* [51]. Bayesian models run through *brms* provide an estimate of the slope (*β*) with 95% credible intervals (CI), which gives an indication as to the effect of the variable in question. If the 95% CIs for the *β* estimate did not overlap with 0, or minimally overlap with 0, which indicates a variable of interest explains some of the variations in the data, we assessed the model’s predictive ability by comparing the model containing the variable of interest with a simpler model that did not contain the variable (or interaction of variables) of interest. We compared these models using leave-one-out (loo) cross-validation and calculated the relative weight of each model to evaluate which model had the highest likelihood of predicting future data [52]. Models fit in this manner move away from the dichotomy of deciding whether a variable is significant or non-significant and assess both the strength of a variable’s effect and the predictive ability of the model. Post hoc contrasts between different pairs were performed using the ‘hypothesis’ function in *brms*.

We tested our predictions by focusing on the presence of a feeding incision on the prepared carrion nest at 53 h after pairing, using data from both the NC and FC post-hatching environments (see figure 1) since parents in both treatments had experienced the same opportunity to create a feeding incision by this point. (Note that when we refer to the ‘NC environment’ and the ‘FC environment’, we mean the within-generation treatment at the end of the experiment (shown in figure 1). We refer to the evolving lines as either the ‘FC lines’ or the ‘NC lines’.) We treated the presence or absence of a feeding incision on the carcass as a dummy variable with a Bernoulli distribution where 0 = no feeding incision and 1 = feeding incision present.

## 3. Results

**Figure 2.**
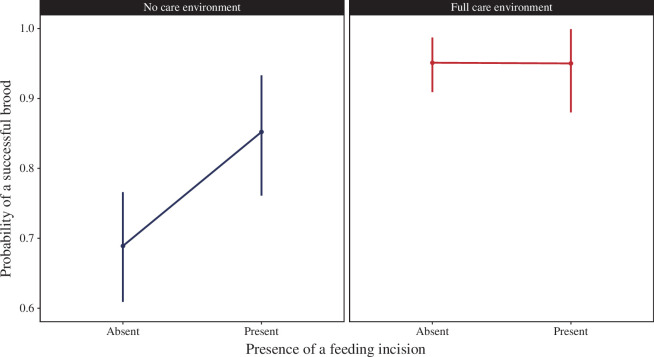
The proportion of successful broods when the feeding incision in the carcass was either present or absent prior to larval hatching, in an NC and FC post-hatching environment. In the NC environment, there were *n* = 138 broods without a feeding incision, and *n* = 60 where the feeding incision was present. In the FC environment, there were *n* = 104 broods with an absent feeding incision, and *n* = 33 with a feeding incision. Note, one pair from the NN pairing in an FC environment was excluded from any analysis involving the presence or absence of the feeding incision as that data were absent. Predicted values (and 95% credible intervals) are shown derived from the model with carcass mass and female and male standardized size as other covariates.

**Figure 3.**
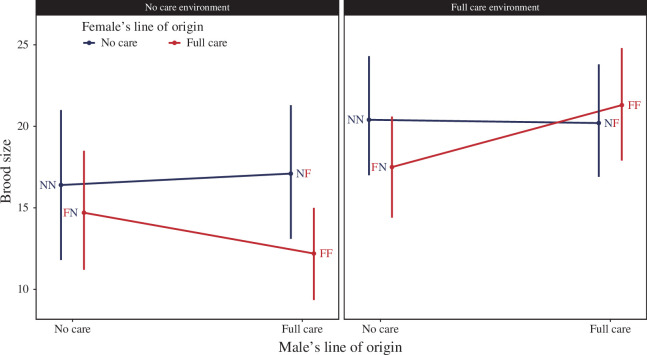
The predicted brood size (and 95% credible intervals) for all pairs across both NC and FC environments. Treatments are listed with female first and male second (e.g. FN is an FC female paired with an NC male), with red letters indicating an FC parent and blue letters indicating an NC parent. Predicted means are shown and are derived from the model containing the lines of origin of the male and the female and their interaction, as well as all other covariates, even if the model with the interaction is not the favoured model (see table 1).

In the NC environment, the presence of a feeding incision before larval hatching increased the chance that the brood produced at least one surviving larva (*β* = 1.02, [0.28, 1.91]; figure 2), though not the number of larvae within a brood (*β* = 0.03, [–0.21, 0.30]). Brood success could not be further explained by any other divergence between the populations of origin, as the null model without female or male background had the lowest loo values and half the model weight (table 1). NC females yielded larger broods in the NC environment, independent of whether her partner was an NC male or an FC male (table 1; figure 3). However, in the FC environment, the presence of a feeding incision did not influence the chance that the brood would produce at least one surviving larva (*β* = 0.21, [–1.53, 2.27]; figure 2), or brood size (= –0.12, [–0.32, 0.09]). The probability of brood success was partly explained by the female’s line of origin, but this model had no more explanatory power than the null model as shown by the small ∆loo and almost equal loo weight. The brood size was not explained by male or female background, with the null model preferred (table 1; figure 3).

**Table 1.** Summary of the model comparisons for brood success (whether a pair produces at least one larva that survives to dispersal) and brood size (the number of dispersing larvae produced given at least one larva survived) in NC and FC environments, and, in the case of the NC environment, whether the inclusion of the feeding incision as an explanatory variable alters which model best explains the data. A ∆loo of 0 indicates it has the lowest looic value, with the difference and standard errors of the difference listed for each model. The looic weight is also listed for each model and sums to 1 for each set of models.

model	brood success		brood success		brood success		brood size		brood size		brood size	
	*No Care with incision*		*No Care without incision*		*Full Care*		*No Care with incision*		*No Care without incision*		*Full Care*	
	∆loo	loo weight	∆loo	loo weight	∆loo	loo weight	∆loo	loo weight	∆loo	loo weight	∆loo	loo weight
female × males	4.67 ± 2.77	0.05	2.21 ± 3.01	0.05	1.72 ± 1.79	0.14	2.39 ± 2.19	0.13	2.26 ± 2.22	0.14	2.76 ± 3.37	0.09
female + male	4.21 ± 1.28	0.06	3.54 ± 1.13	0.08	2.67 ± 1.85	0.09	1.43 ± 1.39	0.21	1.48 ± 1.43	0.20	2.13 ± 2.21	0.13
female	1.67 ± 1.25	0.21	1.89 ± 0.96	0.19	0	0.33	0	0.44	0	0.43	1.55 ± 0.98	0.17
male	2.07 ± 0.37	0.18	1.85 ± 0.86	0.19	1.45 ± 4.50	0.16	3.52 ± 3.87	0.08	3.36 ± 3.86	0.08	0.73 ± 1.96	0.25
null	0	0.50	0	0.49	0.24 ± 3.68	0.29	2.27 ± 3.61	0.14	2.10 ± 3.52	0.15	0	0.36

**Figure 4.**
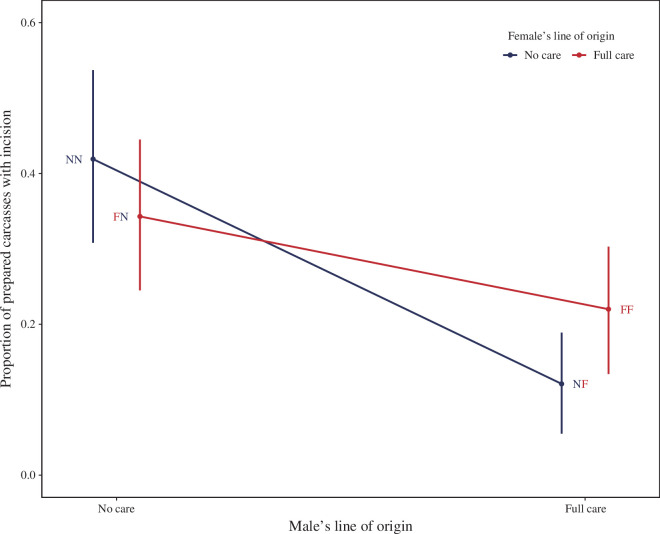
The proportion (and 95% credible intervals that describe the posterior distribution of the estimate) of prepared carrion nests found with an incision hole at 53 h after pairing, in relation to both the male and female’s line of origin. Treatments are listed with female first and male second (e.g. FN is an FC female paired with an NC male), with red letters indicating an FC parent and blue letters indicating an NC parent. Predicted means are shown and are derived from the model containing the lines of origin of the male and the female and their interaction, as well as all other covariates.

Replicating our previous finding [26,36], we found that homotypic NC pairs were more likely to insert a feeding incision by 53 h after pairing than homotypic FC pairs (figure 4; NN versus FF post hoc comparison in table 2). Focusing first on males, we found that a feeding incision was more likely to be made if the male was drawn from an NC population than an FC population (figure 4; table 3). This was true regardless of the female’s line of origin. The NC homotypic pair and the heterotypic pair of an NC male and an FC female did not differ in their likelihood of creating a feeding incision (figure 4; NN versus FN post hoc comparison in table 2). Therefore, we conclude that NC males independently adapted their carcass preparation behaviour in response to the NC treatment, and accelerated their carcass preparation behaviour (to the point of inserting an incision) irrespective of the female they are paired with.

**Table 2.** Post hoc contrasts between all four combinations of female and male lines of origin on the predicted probability of the presence of a feeding incision. The difference between the posterior distributions of the two pairs we are contrasting is shown, summarized by the mean and 90% CIs of the resulting distribution. Treatments are listed with female first and male second (e.g. FN is an FC female paired with an NC male). All comparisons are one-tailed save the FN versus NF comparison. We predicted that the NN would have greatest probability of making an incision and FF having the lowest probability, and so comparisons with these treatments were one-tailed accordingly. We had no *a priori* expectation for FN versus NF, which is why it is a two-tailed comparison. Credible intervals are 90% CIs except for the two-tailed test of FN versus NF which have 95% CIs. The final column identifies which of the two pairs in the contrast is most likely to make the incision, when the modulus of the contrast is greater than 0.

contrast	estimate	**credible intervals**	pair more likely to make incision
FF versus NN	–0.95	[–1.53, –0.36]	NN
FF versus NF	0.74	[0.05, 1.44]	FF
FF versus FN	–0.62	[–1.18, –0.05]	FN
NF versus NN	–1.69	[–2.38, –1.03]	NN
NN versus FN	–0.33	[–0.86, 0.22]	neither
NF versus FN	–2.02	[–3.21, –0.73]	FN

**Table 3.** Summary of the model comparisons for the presence of the feeding incision. A ∆loo of 0 indicates it has the lowest looic value, with the difference and standard errors of the difference listed for each model. The looic weight is also listed for each model and sums to 1 for each set of models.

model	probability of a feeding incision	
	∆loo	loo weight
female × male	0.11 ± 4.21	0.41
female + male	1.84 ± 0.80	0.17
female	17.71 ± 8.65	0.00
male	0	0.42
null	15.56 ± 8.46	0.00

Turning to females, we found evidence that the presence of a feeding incision depended on both the female’s line of origin and the male’s line of origin (figure 4; interaction between sex and line of origin: *β* = 1.06, [–0.02, 2.15]). We found that a feeding incision was more likely to be made when an NC female was paired with an NC male, than when an NC female was paired with an FC male (NN versus NF comparison in table 2). Furthermore, a feeding incision was less likely to be made when an NC female was paired with an FC male than when an FC female was paired with an FC male (NF versus FF comparison in table 2). We conclude that any contributions made by females to incisions in the carrion nest were dependent on the line from which their partner was drawn, and that female contributions were more likely when males and females were drawn from the same line.

## 4. Discussion

We have previously shown that pairs of burying beetles evolved to prepare their carrion nest more rapidly when they were persistently prevented from interacting with their larvae after hatching [29]. Specifically, pairs evolved to hasten their insertion of a feeding incision in the carrion so that it was more likely to be present before their larvae hatched in the NC lines than in the FC lines (figure 4). The presence of a feeding incision contributes significantly to brood fitness when broods are deprived of any post-hatching care, because there is a greater chance that at least one larva survives until dispersal (figure 2) [29,32,36]. Although the combination of male and female background influenced the likelihood of an incision in the carrion nest (table 1; figure 4), it did not contribute directly beyond that to either the chance of brood success or brood size (figure 3). However, interpreting these measures of offspring fitness is not easy and complicated by the fact that the offspring were genetic hybrids between lines and not adapted to the environment in which they developed.

By setting up crosses between our experimental NC and FC lines, we dissected the evolution of carrion preparation behaviour to determine how individual changes in males and females contributed to the accelerated expression of this pair-level trait in the NC lines. We found that NC males had evolved to ensure the feeding incision was inserted sooner into the carcass nest and that this adaptation was expressed independent of the female’s line of origin. One possible inference is that males independently adapt the pace of their carrion nest preparation to the wider ecological conditions in which they live in the wild, such as the risk of carrion takeover by rivals, and are insensitive to the behaviour of their partner.

By contrast, from the female’s perspective, the likelihood that a feeding incision would be made prior to larval hatching was much more contingent on the line from which her partner was drawn. From the NC female’s perspective, an incision was more likely to be made when her partner came from an NC line too rather than an FC line. Likewise, from the FC female’s perspective, an incision was more likely to be made when her partner was from an FC line than from an NC line. We draw two inferences here. First, NC females coadapted their behaviour to NC male carrion nest preparation behaviour—though we cannot tell from the data we collected exactly how this coadaptation was expressed. Second, it is also possible that females also plastically adjusted their behaviour in relation to male behaviour, though evidently there were limits on the extent of female plasticity because otherwise we would not have found any contingency on the male line of origin.

In future work, it would be interesting to measure individual-level reaction norms for the female’s incision-making behaviour in response to their social partner, to assess if such a behaviour is contingent on social partner and, if so, whether the extent of plasticity evolved during the course of the experiment.

The strength of our conclusions is tempered by the fact that our experimental design lacked any control pairings within replicate lines (we had to sacrifice this treatment owing to logistical constraints). Nevertheless, from other data we have collected from these populations, we have no reason to suppose that the replicate lines, within either the NC or FC treatments, had diverged by this stage of experimental evolution [29,34–38] (but see [53]). If our conclusions are broadly correct, then our expectation is that the evolution of other group or pair-level traits in response to environmental change should be initiated unilaterally by the individual that takes that lead in shaping these collective actions, and that this in turn will provoke swift social coadaptation by other members of the pair or group.

The coadaptation of male and female traits is likely to have favoured a distinct combination of socially interacting genes in the two sexes (cf. [19]). For this reason, previous theoretical analyses have suggested that socially coadapted traits within the family that are divergent between populations could function as a post-mating mechanism for reproductive isolation—because they cause hybrids to perform less well (e.g. [18,54–56]). A recent speciation event within the *Nicrophorus* genus may help better understand how post-mating barriers imposed by parental care could contribute to speciation. *Nicrophorus vespilloides* has recently been split into two species, with *N. hebes* now recognized as a distinct bog-breeding specialist that lives mostly in Canada [57]. Speciation is sufficiently recent that *N. hebes* can still hybridize with Alaskan populations of *N. vespilloides* to produce viable offspring. Nevertheless, hybrids perform less well, partly because hybrid larvae are less viable (at least in an NC post-hatching environment). Experimental work indicates that hybrid pairings produce fewer eggs and have lighter broods than pure-bred populations [57]. There are several reasons why hybrid pairings have lower success, but the experiment we present here suggests that it could be partly owing to divergence in the extent of coadaptation between care-giving adults. We know that *N. hebes* and *N. vespilloides* differ in the ecological environments they inhabit in the wild, which could affect predation risk, the abundance of carcasses available as breeding resources, or the intensity of competition for carcasses from congeneric species [58], and therefore the relative costs and benefits of parents staying with their offspring after hatching. This, in turn, could influence the probability that broods will be left to develop without their parents, causing divergence between wild populations that mimic the FC and NC conditions we created experimentally in the lab. If populations then diverge in the ways that males and females are coadapted during biparental care, as we report here, then hybrid pairings will be less successful. However, whether a mechanism like this can explain the low breeding success of *N. hebes* and *N. vespilloides* hybrids remains to be tested explicitly.

Social coadaptations contribute to post-mating reproductive isolation in other species (e.g. [18,54–56]). The key barrier is the high level of coordination between interacting individuals. This is true whether coordination results from cooperation or conflict. It is seen in viviparous species, for example, through the highly specific structures that have evolved to coordinate the supply of resources from mother to offspring in viviparous fish [59,60] and placental mammals [61].

We have shown here that the highly coordinated activities jointly undertaken by male and female burying beetles in converting carrion into an edible nest can also become coadapted and cause heterotypic pairings to perform less well. In future work, it would be interesting to test whether the coadaptations involved in other types of coordinated cooperative social activities, such as the construction of nests or burrows, collective immunity, or the joint defence of a key resource, could also potentially function as a post-mating barrier to gene flow between populations.

## Data Availability

Data are available from Dryad Digital Repository [62].

## References

[1] Chapman T, Arnqvist G, Bangham J, Rowe L. 2003 Sexual conflict. Trends Ecol. Evol. **18**, 41–47. (10.1016/S0169-5347(02)00004-6)

[2] Royle NJ, Smiseth PT, Kölliker M. 2012 The evolution of parental care. Oxford, UK: Oxford University Press. (10.1093/acprof:oso/9780199692576.001.0001)

[3] Emlen ST. 1995 An evolutionary theory of the family. Proc. Natl Acad. Sci. USA **92**, 8092–8099. (10.1073/pnas.92.18.8092) 7667250 PMC41102

[4] Queller DC, Strassmann JE. 2009 Beyond society: the evolution of organismality. Phil. Trans. R. Soc. B **364**, 3143–3155. (10.1098/rstb.2009.0095) 19805423 PMC2781869

[5] Cornwallis CK, Botero CA, Rubenstein DR, Downing PA, West SA, Griffin AS. 2017 Cooperation facilitates the colonization of harsh environments. Nat. Ecol. Evol. **1**, 0057. (10.1038/s41559-016-0057) 28812731

[6] Feeney WE, Medina I, Somveille M, Heinsohn R, Hall ML, Mulder RA, Stein JA, Kilner RM, Langmore NE. 2013 Brood parasitism and the evolution of cooperative breeding in birds. Science **342**, 1506–1508. (10.1126/science.1240039) 24357317

[7] Queller DC, Strassmann JE. 1998 Kin selection and social insects. Bioscience **48**, 165–175. (10.2307/1313262)

[8] Faulkes CG, Bennett NC, Bruford MW, O’Brien HP, Aguilar GH, Jarvis JUM. 1997 Ecological constraints drive social evolution in the African mole–rats. Proc. R Soc. B **264**, 1619–1627. (10.1098/rspb.1997.0226) PMC1688729 9404025

[9] Hansell M. 2005 Animal architecture. Oxford, UK: Oxford University Press. (10.1093/acprof:oso/9780198507529.001.0001)

[10] Cremer S. 2019 Social immunity in insects. Curr. Biol. **29**, R458–R463, (10.1016/j.cub.2019.03.035) 31163158

[11] Barta Z, Székely T, Liker A, Harrison F. 2014 Social role specialization promotes cooperation between parents. Am. Nat. **183**, 747–761. (10.1086/676014) 24823819

[12] Cooper GA, West SA. 2018 Division of labour and the evolution of extreme specialization. Nat. Ecol. Evol. **2**, 1161–1167. (10.1038/s41559-018-0564-9) 29807994

[13] Itzkowitz M, Santangelo N, Richter M. 2001 Parental division of labour and the shift from minimal to maximal role specializations: an examination using a biparental fish. Anim. Behav. **61**, 1237–1245. (10.1006/anbe.2000.1724)

[14] McNamara JM, Gasson CE, Houston AI. 1999 Incorporating rules for responding into evolutionary games. Nature **401**, 368–371. (10.1038/43869) 10517633

[15] Moore AJ, Brodie ED, Wolf JB. 1997 Interacting phenotypes and the evolutionary process. I. Direct and indirect genetic effects of social interactions. Evolution **51**, 1352–1362. (10.1111/j.1558-5646.1997.tb01458.x) 28568644

[16] Agrawal AF, Brodie ED, Wade MJ. 2001 On indirect genetic effects in structured populations. Am. Nat. **158**, 308–323. (10.1086/321324) 18707327

[17] Drown DM, Wade MJ. 2014 Runaway coevolution: adaptation to heritable and nonheritable environments. Evolution. **68**, 3039–3046. (10.1111/evo.12470) 24916074 PMC4184967

[18] Zeh DW, Zeh JA. 2000 Reproductive mode and speciation: the viviparity-driven conflict hypothesis. Bioessays **22**, 938–946. (10.1002/1521-1878(200010)22:10<938::AID-BIES9>3.0.CO;2-9) 10984720

[19] Linksvayer TA, Fondrk MK, Page Jr. RE. 2009 Honeybee social regulatory networks are shaped by colony‐level selection. Am. Nat. **173**, E99–E107. (10.1086/596527) 19140771

[20] Hinde CA, Johnstone RA, Kilner RM. 2010 Parent-offspring conflict and coadaptation. Science **327**, 1373–1376. (10.1126/science.1186056) 20223985

[21] Savage JL, Hinde CA. 2019 What can we quantify about carer behavior? Front. Ecol. Evol. **7**, 418. (10.3389/fevo.2019.00418)

[22] Griffith SC. 2019 Cooperation and coordination in socially monogamous birds: moving away from a focus on sexual conflict. Front. Ecol. Evol. **7**, 455. (10.3389/fevo.2019.00455)

[23] Smiseth PT. 2019 Coordination, cooperation, and conflict between caring parents in burying beetles. Front. Ecol. Evol. **7**, 397. (10.3389/fevo.2019.00397)

[24] Wolf JB, Brodie ED, Wade MJ. 2000 Epistasis and the evolutionary process. New York, NY: Oxford University Press.

[25] Wolf JB. 2000 Indirect genetic effects and gene interactions. In Epistasis and the evolutionary process (eds JB Wolf, ED Brodie, MJ Wade), pp. 158–176. Oxford, UK: Oxford University Press.

[26] Nagy M, Akos Z, Biro D, Vicsek T. 2010 Hierarchical group dynamics in pigeon flocks. Nature **464**, 890–893. (10.1038/nature08891) 20376149

[27] Hager R, Johnstone RA. 2003 The genetic basis of family conflict resolution in mice. Nature **421**, 533–535. (10.1038/nature01239) 12556892

[28] Kölliker M, Royle NI, Smiseth PT. 2012 Parent–offspring co-adaptation. In The evolution of parental care (eds NJ Royle, PT Smiseth, M Kölliker), pp. 285–303. Oxford, UK: Oxford University Press. (10.1093/acprof:oso/9780199692576.001.0001)

[29] Duarte A, Rebar D, Hallett AC, Jarrett BJM, Kilner RM. 2021 Evolutionary change in the construction of the nursery environment when parents are prevented from caring for their young directly. Proc. Natl Acad. Sci. USA **118**, e2102450118. (10.1073/pnas.2102450118) 34819363 PMC8640939

[30] Scott MP. 1998 The ecology and behavior of burying beetles. Annu. Rev. Entomol. **43**, 595–618. (10.1146/annurev.ento.43.1.595) 15012399

[31] Müller JK, Braunisch V, Hwang WB, Eggert AK. 1998 Alternative tactics and individual reproductive success in natural associations of the burying beetle, Nicrophorus vespilloides. Behav. Ecol **18**, 196–203. (10.1093/beheco/arl073)

[32] Eggert A-K, Reinking M, Muller JK. 1998 Parental care improves offspring survival and growth in burying beetles. Anim. Behav. **55**, 97–107. (10.1006/anbe.1997.0588) 9480676

[33] Sun S-J, Kilner RM. 2020 Temperature stress induces mites to help their carrion beetle hosts by eliminating rival blowflies. Elife **9**, e55649. (10.7554/eLife.55649) 32755542 PMC7431131

[34] Schrader MS, Jarrett BJM, Kilner RM. 2015 Using experimental evolution to study adaptations for life within the family. Am. Nat. **185**, 610–619. (10.1086/680500) 25905504 PMC4497813

[35] Schrader M, Jarrett BJM, Rebar D, Kilner RM. 2017 Adaptation to a novel family environment involves both apparent and cryptic phenotypic changes. Proc. R. Soc. B **284**, 20171295. (10.1098/rspb.2017.1295) PMC5597835 28878064

[36] Jarrett BJM, Evans E, Haynes HB, Leaf MR, Rebar D, Duarte A, Schrader M, Kilner RM. 2018 A sustained change in the supply of parental care causes adaptive evolution of offspring morphology. Nat. Commun. **9**, 3987. (10.1038/s41467-018-06513-6) 30266903 PMC6162320

[37] Jarrett BJM, Rebar D, Haynes HB, Leaf MR, Halliwell C, Kemp R, Kilner RM. 2018 Adaptive evolution of synchronous egg-hatching in compensation for the loss of parental care. Proc. R. Soc. B **285**, 20181452. (10.1098/rspb.2018.1452) PMC6125895 30158310

[38] Rebar D, Bailey NW, Jarrett BJM, Kilner RM. 2020 An evolutionary switch from sibling rivalry to sibling cooperation, caused by a sustained loss of parental care. Proc. Natl Acad. Sci. USA **117**, 2544–2550. (10.1073/pnas.1911677117) 31964847 PMC7007579

[39] Rebar D, Halliwell C, Kemp R, Kilner RM. 2022 Experimental evolution of a more restrained clutch size when filial cannibalism is prevented in burying beetles Nicrophorus vespilloides. Ecol. Evol. **12**, e8829. (10.1002/ece3.8829) 35441005 PMC9012908

[40] Henshaw JM, Fromhage L, Jones AG. 2019 Sex roles and the evolution of parental care specialization. Proc. R. Soc. B **286**, 20191312. (10.1098/rspb.2019.1312) PMC6732396 31455191

[41] Walling CA, Stamper CE, Smiseth PT, Moore AJ. 2008 The quantitative genetics of sex differences in parenting. Proc. Natl Acad. Sci. USA **105**, 18430–18435. (10.1073/pnas.0803146105) 19008350 PMC2587554

[42] De Gasperin O, Duarte A, Troscianko J, Kilner RM. 2016 Fitness costs associated with building and maintaining the burying beetle’s carrion nest. Sci. Rep. **6**, 35293. (10.1038/srep35293) 27734965 PMC5062497

[43] Pilakouta N, Hanlon EJH, Smiseth PT. 2018 Biparental care is more than the sum of its parts: experimental evidence for synergistic effects on offspring fitness. Proc. R. Soc. B **285**, 20180875. (10.1098/rspb.2018.0875) PMC6111165 30068674

[44] R Development Core Team. 2021 *R: a language and environment for statistical computing. 3.5.1*. Vienna, Austria. R Foundation for Statistical Computing.

[45] Stan Development Team. 2021 *Stan modeling language users guide and reference manual*.2.19.1. See https://mc-stan.org.

[46] Bürkner PC. 2017 brms: an R package for Bayesian multilevel models using stan **80**, 1–28. (10.18637/jss.v080.i01)

[47] Bürkner P-C. 2018 Advanced Bayesian multilevel modeling with the R package brms. R J. **10**, 395–411. (10.32614/RJ-2018-017)

[48] Wickham H. 2017 *tidyverse: easily install and load the 'Tidyverse*’. R package version 1.2.1. See https://CRAN.R-project.org/package=tidyverse.

[49] Wickham H. 2009 *ggplot2: elegant graphics for data analysis*. New York, NY: Springer. (10.1007/978-0-387-98141-3)

[50] Kay M. 2019 *tidybayes: tidy data and geoms for Bayesian models*. R package version 482 1.1.0. (10.5281/zenodo.130815)

[51] Wickham H. 2018 *modelr: modelling functions that work with the pipe*. R package version 0.1.2. See https://CRAN.R-project.org/package=model.

[52] Vehtari A, Gelman A, Gabry J. 2017 Practical Bayesian model evaluation using leave-one-out cross-validation and WAIC. Stat. Comput. **27**, 1413–1432. (10.1007/s11222-016-9696-4)

[53] Bladon EK, Pascoal S, Kilner RM. 2022 The role of recent evolutionary history in resilience to environmental change: social evolution effects versus founder effects. bioRxiv (10.1101/2022.11.04.515151) PMC11486917 39431165

[54] Gavrilets S. 2000 Rapid evolution of reproductive barriers driven by sexual conflict. Nature **403**, 886–889. (10.1038/nature01752) 10706284

[55] Martin OY, Hosken DJ. 2003 The evolution of reproductive isolation through sexual conflict. Nature **423**, 979–982. (10.1038/nature01752) 12827200

[56] Brandvain Y, Haig D. 2005 Divergent mating systems and parental conflict as a barrier to hybridization in flowering plants. Am. Nat. **166**, 330–338. (10.1086/432036) 16224688

[57] Sikes DS, Trumbo ST, Peck SB. 2016 Cryptic diversity in the new world burying beetle fauna: Nicrophorus hebes Kirby; new status as a resurrected name (Coleoptera: Silphidae: nicrophorinae). Arthropod Syst. Phylogenet. **74**, 299–309. (10.1002/evl3.176)

[58] Sun SJ, Catherall AM, Pascoal S, Jarrett BJM, Miller SE, Sheehan MJ, Kilner RM. 2020 Rapid local adaptation linked with phenotypic plasticity. Evol. Lett. **4**, 345–359. (10.1002/evl3.176) 32774883 PMC7403679

[59] Schrader M, Travis J. 2008 Testing the viviparity‐driven‐conflict hypothesis: parent‐offspring conflict and the evolution of reproductive isolation in a poeciliid fish. Am. Nat. **172**, 806–817. (10.1086/592999) 18950276

[60] Furness AI, Pollux BJA, Meredith RW, Springer MS, Reznick DN. 2019 How conflict shapes evolution in poeciliid fishes. Nat. Commun. **10**, 1–12. (10.1038/s41467-019-11307-5) 31350395 PMC6659687

[61] Roy SW. 2022 Haldane’s duel: intragenomic conflict, selfish Y chromosomes and speciation. Trends Genet. **38**, 8–11. (10.1016/j.tig.2021.05.008) 34167831

[62] Jarrett BJM, Mashoodh R, Issar S, Pascoal S, Rebar D, Sun SJ, Schrader M, Kilner RM. 2024 Data for: selection on the joint actions of pairs leads to divergent adaptation and coadaptation of care-giving parents during pre-hatching care. Dryad Digital Repository (10.5061/dryad.2v6wwpzw) PMC11285745 38864319

